# Misaligned hope and conviction in health care

**DOI:** 10.1111/bioe.13370

**Published:** 2024-11-16

**Authors:** Steve Clarke, Justin Oakley, Jonathan Pugh, Dominic Wilkinson

**Affiliations:** ^1^ School of Social Work and Arts Charles Sturt University Wagga Wagga New South Wales Australia; ^2^ Uehiro Oxford Institute University of Oxford Oxford UK; ^3^ Monash Bioethics Centre Monash University Clayton Victoria Australia; ^4^ Uehiro Oxford Institute, John Radcliffe Hospital University of Oxford Oxford UK; ^5^ Murdoch Children's Research Institute, Centre for Biomedical Ethics National University of Singapore Singapore

**Keywords:** autonomy, conviction, decision‐making capacity, health care, hope, practical rationality

## Abstract

It is often said that it is important for patients to possess hope that their treatment will be successful. We agree, but a widely appealed to type of hope—hope based on conviction (religious or otherwise), renders this assertion problematic. If conviction‐based hope influences patient decisions to undergo medical procedures, then questions are raised about the scope of patient autonomy. Libertarians permit patients to make decisions to undergo medical procedures on the basis of any considerations, including conviction‐based hopes, on grounds of respect for freedom of choice. Rational interventionists want to restrict choices made on the basis of conviction‐based hope on the grounds that choices based on hope incorporate irrationality of a sort incompatible with autonomous decision‐making. In this article, we navigate a middle path between these extremes, arguing that patient decision‐making based on conviction‐based hope ought to be acceptable and permitted in health care when it conforms to norms of practical rationality. These norms allow patients some room to make decisions to consent to undergo medical procedures informed by conviction‐based hope.

## 1 SECTION 1

It is often said that it is important that patients undergoing healthcare procedures should possess hope that their treatment will be successful.^1^ Many bioethicists hold that healthcare professionals should try, where possible, to foster and support such hope in their patients.^2^ A key reason for this (which we accept) is that hope has motivating qualities.^3^ A patient who possesses hope of successful treatment will be motivated to cooperate during a healthcare procedure, to take medication, undertake programs of exercise, and stick to any prescribed dietary restrictions, etc., which may be required of them. By contrast, a patient who lacks hope of a successful treatment may not be motivated to cooperate, do what is required to complete a successful rehabilitation program, and/or restrict their diet. All things being equal, the patient who possesses hope of a successful treatment will attain better healthcare outcomes than the patient who lacks such hope.^4^

When they encourage patients to hope for successful healthcare outcomes, healthcare professionals risk encouraging ‘false hope’: that is hope that is not based on a realistic understanding of the chances of success and failure of a particular healthcare procedure.^5^ There are two key reasons why it is problematic if patients possess false hope of a successful healthcare outcome. The first is that the standard informed consent process requires patients sufficiently to understand the risks involved in any healthcare procedure, before that procedure can be permitted to take place.^6^ If patients are deemed to be unable to understand the chances of success and failure of a particular healthcare procedure, then they cannot provide ‘valid consent’ to that procedure. We will have more to say about valid consent in Section 4. The second is that patients need to be prepared for the possibility of an unsuccessful outcome.^7^ A patient with false hope who consequently fails to consider the chance of a healthcare procedure being unsuccessful is less likely to make any needed preparations for this eventuality, including the possibility of their death, if that is a potential outcome of a particular unsuccessful healthcare procedure. A patient with hope based on a realistic understanding of the chances of success and failure of a particular healthcare procedure would ‘hope for the best but prepare for the worst,’ as Benjamin Disraeli famously advised.^8^

Anecdotal evidence suggests that it is not uncommon for there to be misalignment between patients' hopes and realistic assessments of the chances of a particular healthcare procedure succeeding or failing, as advised to those patients by health professionals.^9^ In this article, we will consider what healthcare professionals can do when a patient's convictions (religious or otherwise) leads them to either have unrealistic expectations of the chances of a successful treatment or to have hopes that are aimed at different goals than the ordinary goals of health care.^10^ A possible response for some healthcare professionals who have a high level of understanding of the sources of their patients' convictions might be to reason with those patients and help them to replace their unrealistic conviction‐based hopes and expectations with realistic evidence‐based ones. However, we recognise that many healthcare professionals may be ill‐equipped to attempt such an act of replacement and that it may be impossible to achieve this anyway. Conviction (religious or otherwise) is often not something that gives way in the face of appeals to evidence. We aim to generate conceptual clarification and provide practical ethical advice to healthcare professionals who find themselves dealing with patients whose conviction‐based hopes and expectations are out of kilter with the hopes and expectations that would help facilitate the attainment of the ordinary goals of health care.

The remainder of the article will be organised as follows: In the next section, we will clarify how we understand key terms. In the section after that, we will describe several cases in which conviction leads patients to hope to diverge from the goals and expectations that healthcare professionals recommend. In Section 4, we will examine some theoretical considerations that healthcare professionals need to consider when responding to the cases described. Our focus is on ethical considerations, but for context we will mention the way in which the law (using the example of the England and Wales) addresses the issues under discussion. We will consider how healthcare professionals can respect patient autonomy when the unusual hopes that some patients express raise questions about the degree to which their consent to participation in particular healthcare procedures can be autonomous. In Section 5, we will recommend and discuss practical responses to the cases described. The paper ends with a brief concluding remark, summarising our findings.

Our overall approach to our chosen topic is to navigate a middle path between two unsatisfactory extremes. One extreme is a libertarian approach that seeks to respect the freely made choices of patients based on hope, regardless of the rationality of such decisions, even when these are against the best interests of patients. The other extreme is a strongly rationalist interventionist approach that claims that decisions grounded in hope do not warrant respect because hope incorporates irrationality of a sort that is incompatible with autonomous decision‐making, on rationalist views of autonomy.^11^ On this rationalist interventionist approach, many patient decisions based on hope would not be respected because they are not understood to be autonomous. We will refer to our preferred approach as the ‘practical rational’ view, for reasons that will become clear in Section 4.

## 2 SECTION 2

For the purposes of this article, we will understand *conviction* broadly to include firmly and seriously held beliefs whose truth has not been established on the basis of empirical evidence or rational argument. On our account, this includes ordinary forms of religious belief grounded in religious faith, but also belief in methods of health care that lack a conventional evidential basis. Someone who holds the conviction that the influence of the healing power of crystals can cure disease may well not be guided by a fully‐fledged set of beliefs, akin to a religion. Nevertheless, it seems clear to us that such evidence‐independent beliefs share sufficient features with religious convictions that can give rise to hope which can come into conflict with the ordinary aims of health care. They can do so by leading people to hope for the success of forms of treatment that conventional medicine does not endorse and by leading people to accept assessments of the likelihood of the success of ordinary forms of treatment that are in conflict with the assessments of conventional healthcare professionals.

We will work with Adrienne Martin's influential definition of hope, which helpfully relates *hope* to specific outcomes (such as a successful treatment). According to her:

to hope for an outcome is to *desire* (be attracted to) it, to assign a *probability* somewhere between 0 and 1 to it, and to judge that there are sufficient *reasons* to engage in certain feelings and activities directed toward it.^12^

Martin's definition of hope incorporates a cognitive component (pertaining to the assignment of a probability to the hoped‐for outcome) and a non‐cognitive component (pertaining to the practical reasons that one has to adopt positive *attitudes* towards the outcome).

We understand the *ordinary aim of health care* to involve restoring patients to states of good health, where this is possible.^13^ In circumstances where a patient cannot be returned to a state of health and is expected to die soon, the ordinary aim of healthcare shifts to become a palliative goal of supporting the patient and their family for the patient's remaining lifespan.^14^ These are the goals that ordinary contemporary healthcare professionals aim for. We do not understand the aims of health care to include any form of human enhancement or life extension beyond what is naturally possible. Nor do we understand the aims of health care to involve concern with the state of any possible afterlife that a patient might or might not go on to attain after their lives have ended. We have no objection to people pursuing such aims, but they cannot expect the assistance of healthcare professionals, who are not trained to assist them in achieving such aims.

Situations where patient hopes are aligned with the expectations presented to patients by healthcare professionals, and which lead those patients to aim for outcomes that the healthcare professionals treating them recommend, raise few if any problems and are not of interest to us in this article. Problems can arise when patients hope for outcomes that diverge from those recommended by healthcare professionals or have hopes for recommended outcomes that are significantly stronger (or significantly fainter) than are warranted, given the prospects for successful treatment presented to them by the healthcare professionals treating them.

## 3 SECTION 3

Here are four different ways that the hopes of patients and health professionals may be misaligned. They are: (a) when conviction leads patients to hope for outcomes that conventional medicine holds to be unattainable, (b) when conviction leads patients to have unrealistic expectations of success and to discount the possibility that medical treatment may fail, (c) when conviction leads patients to hope for outcomes that are attainable, but which are not the preferred outcomes of practitioners of conventional medicine, and (d) when conviction leads patients to have unrealistically faint hopes of success and to be insufficiently motivated to fully co‐operate during a healthcare procedure and a subsequent rehabilitation program.^15^

(a) When conviction leads patients to hope for outcomes that conventional medicine holds to be unattainable.

Marta is 35 weeks pregnant with a baby girl. During pregnancy, repeated ultrasounds demonstrated that the baby had very little fluid around her and appeared to have no kidneys (a problem called renal agenesis). This is an uncommon but extremely serious problem arising early in the development of a baby. Once born, it will have severe problems relating to renal and respiratory failure and is likely to die within a very short period after birth. Without fluid around the baby, the fetal lungs do not develop. Marta is informed of all of this and understands the information provided by her doctors. However, she has a persistent hope that when her baby is born, the scans will turn out to be mistaken and the baby will be fine. When asked about this belief, she refers to the story of a friend of the family who had been told of serious problems in their baby (and encouraged to terminate), but who was healthy when born. Marta declines opportunities to meet with palliative care specialists or to talk about the care of her baby after birth because she ‘is trying to stay hopeful’.^16^

(b) When conviction leads patients to have unrealistically high hopes of success and to discount the possibility that medical treatment may fail.

A 19‐year‐old British woman, ST, with a rare progressively degenerative mitochondrial disorder, had spent a year in an intensive care unit and is mechanically ventilated. Her doctors believed that she had no prospect of recovery and was fast approaching the end of her life. ST (who despite her severe illness was able to communicate) did not believe the prognosis communicated by the doctors and had an ‘unshakeable belief’ in her ability to survive and recover.^17^ She did not accept that her death was imminent and expressed hope that she would be able to move to North America and attempt to enrol in a clinical trial for an experimental treatment that could potentially offer her an improved quality of life, even if the trip across the Atlantic was risky for her and the experimental treatment could not cure her disorder. However, participation in the trial could not provide any immediate benefits because it had been paused due to funding restraints. ST's reasoning was informed by ‘both her religious faith and the love and support of her family’. ST appealed to the England and Wales Court of Protection to be allowed to pursue her preferred course of action, however, the presiding judge declined her appeal. The judge concluded that although ST comprehended the nature of her disorder, she lacked the capacity to decide about her preferred course of action because she clung ‘… to the hope that her doctors are wrong’ and was incapable of rationally responding to the extremely high objective chance that her preferred course of action would fail.

(c) When conviction leads patients to hope for outcomes that conventional medicine holds to be attainable, but which are not the preferred outcomes of practitioners of conventional medicine.

A 46‐year‐old Amish man presented at the McMaster University burns unit, having sustained burns of a mixed depth to 22% of his total body surface area. The surgeons attending the Amish man recommended conventional medical treatments for burns as well as skin grafts on the more severely burned parts of the man's body. The Amish man declined such treatments as he and the elders within his community regarded these as inconsistent with Amish religious beliefs. Because of his religious beliefs, he was unconcerned by the prospects of lasting scarring on his skin. Rather than hoping to reduce scarring and have his skin restored to something approximating its earlier unburned state – as per the aims of conventional burns treatment – he hoped to return to work as quickly as possible, while also minimising costs. Instead of turning him away, the surgical unit kept him in their care while allowing Amish elders to apply traditional herbal ointments to his wounds and to wrap them in a burdock leaf dressing (known to have anti‐inflammatory, antimicrobial, and analgesic properties^18^) that was changed every twelve hours. The elders also agreed to allow the man to be treated with conventional antibiotics, which proved effective.^19^ The burns unit staff went along with the Amish man's hoped‐for goals and set aside the ordinary aim of reducing scarring, aiming instead to help enable their patient's wounds to heal to the extent that he could return to work safely, while also respecting the cultural and religious values of the Amish community of Ontario, Canada.^20^

(d) When conviction leads patients to have unrealistically faint hopes of success and to be insufficiently motivated to fully co‐operate during a healthcare procedure and a subsequent rehabilitation program.

Drew and Schoenberg report on a number of cases of women in Appalachian Kentucky with cervical cancer for whom hysterectomy was recommended and who delayed treatment because of fatalistic beliefs about the spread of cancer.^21^ One of the cases they report was Helen who was diagnosed with cervical cancer at age 48, and who put up with chronic uterine bleeding for 15 years before agreeing to have a hysterectomy.^22^ It seems that such delays were driven by a combination of economic considerations alongside fatalistic religious beliefs that led the Appalachian women to form unrealistically faint hopes of a successful treatment. Helen and other such patients reported their conviction that a healthcare intervention would do little or nothing to influence the chances of their cancer spreading because, ‘It's in God's hands—not mine.’^23^ Helen only consented to be operated on when abdominal pain and internal bleeding became unbearable and not because she was persuaded that a hysterectomy could make a difference to the chances of cancer spreading to other parts of her body.^24^

## 4 SECTION 4

It is widely accepted that under typical circumstances, it is permissible to perform a medical procedure on a patient with decision‐making capacity only when they have given valid consent to that intervention. Following Beauchamp and Childress, we may say that valid consent amounts to an autonomous authorisation of a medical treatment made intentionally, in the absence of controlling influence, and on the basis of sufficient understanding of information material to the decision.^25^ Autonomous refusals of treatment must meet similar conditions. To have the capacity to make such autonomous treatment decisions, a patient requires the abilities necessary to making decisions in accordance with the aforementioned conditions. This includes the ability to understand information material to the decision.

One of the thorny questions pertaining to consent in medical ethics concerns how the criterion of understanding should be interpreted. We lack the space to substantially enter into debate about the answer here, but it is worth sketching out two possible views. On a ‘thin view,’ the understanding that autonomous decision‐making requires involves only that the individual comprehends certain discrete propositions (e.g., that a surgical procedure will require them to undergo general anaesthesia). On this sort of view, patients need only understand relevant statistical information about the probability of a treatment outcome; they do not need to appreciate that this probability also applies to their own case.^26^

On a thicker view, the criterion of understanding further requires that the individual appreciates that material information applies to their own case and that their decision‐making, based on these beliefs, accords with norms of epistemic rationality pertaining to belief. To illustrate the difference, consider Savulescu and Momeyer's hypothetical case of a patient who reasons in the following way:

(1) There is a risk of dying from anaesthesia. (true)
(2) I will require an anaesthetic if I am to have this operation. (true)

Therefore, if I have this operation, I will probably die.^27^

Does a patient reasoning in this way have sufficient understanding? On a thin view of understanding, the answer is ‘yes’; after all, the patient comprehends the material information outlined in statements (1) and (2). However, on a thicker view, the patient lacks sufficient understanding because their reasoning fails to abide by norms of epistemic rationality. The conclusion the patient draws does not follow from the premises that they comprehend.

The motivation for adopting the thicker view is that it better captures the understanding that is required for autonomous decision‐making. As Savulescu and Momeyer put it: ‘we cannot form an idea of what we want without knowing what the options on offer are like.’^28^ We can fail to know what our options are like because we are ignorant of certain information, we can fail because we do not appreciate that the information we have applies to our own case, and we can fail if we do not use the information we have in compliance with basic norms that govern the rationality of beliefs. For this reason, many theorists have supported the claim that the understanding that autonomy involves a significant degree of appreciation,^29^ whilst others have suggested that irrational beliefs are inimical to autonomous decision‐making.^30^

We will return to the implications of these interpretations of understanding below. First though, let us focus on the broader question that we are concerned with in this article, namely, the implications of hope in medical treatment decisions. When patients lack capacity, one widely endorsed approach asserts that decisions made on their behalf should be in accordance with an assessment of their best interests.^31^ The specific decisions made in our exemplar cases of conviction‐based hope raise questions about both the capacity of decision‐makers to provide valid consent (as was challenged in ST's case), and whether treatment is in their best interests. Although the hope‐based beliefs and values of the patients in question need to be taken seriously in a best interests assessment, such hopes may run contrary to other factors that need to be considered in an overall assessment of their best interests. Such factors will include a physician's professional verdict about the likely medical outcomes that follow from a decision. How such factors ought to be weighed in an overall best interests assessment is a complex question.

Equally complex questions arise with respect to the implications of hope for autonomy and consent. As detailed above, an autonomous treatment decision needs to be based on a sufficient understanding of information that is material to that decision. But, in so far as hope is grounded in non‐rational beliefs, it can be inimical to the understanding that autonomous decision‐making requires—a point that the rationalist interventionist stresses and that we explore further below. However, respect for autonomy requires that patients be permitted to make treatment decisions in accordance with their own values and that medical professionals demonstrate respect for conviction‐based belief—a point that the libertarian makes much of. How can these competing considerations be reconciled?

The law, in England and Wales at least, offers one practical solution to this question. The Mental Capacity Act 2005 (MCA) states that a patient may be found to lack the capacity to make their own treatment decisions only if two tests are passed.^32^ First, the person must be shown to lack an ability that renders them unable to make a decision for themselves, such as the ability to understand material treatment information. Second, that inability must be attributed to an ‘impairment of, or disturbance in, the mind or the brain.’^33^ So, even if we assume that some forms of hope are inimical to the understanding that autonomous decision‐making requires, that alone is potentially not sufficient to justify the claim that their treatment decisions lack legal authority on grounds of a lack of capacity.^34^

From a legal perspective at least, establishing that a patient's hope is irrational may thus not be a sufficient ground to overrule their choice. Nonetheless, the implications of hope warrant further philosophical inquiry, given the deeper conceptual questions they raise for considerations of both autonomy and beneficence. With these implications in mind, let us consider the issues that arise in the cases outlined in Section 3. Consider first cases (a), (b), and (d). These are examples in which an individual's assignment of a probability to an outcome is not rationally warranted given the available evidence. If hope is distorting deliberative processes, does this imply that the patients lack the understanding required for autonomous decision‐making, as the rationalist interventionist tells us? One might deny this claim if one adopts the thin conception of understanding discussed above. On this thin view, physicians are obliged to disclose relevant material information and to ensure that patients sufficiently understand it; but they are not obliged to intervene further when a patient disregards that information because of hope.

In contrast, if one accepts the thicker view of understanding, then it can be argued that the physician's role in the decision‐making process incorporates an obligation to remedy failures of theoretical rationality in the patient's deliberative process. Indeed, Savulescu and Momeyer argue that physicians ought to ‘help their patients to deliberate more effectively and to care more about thinking rationally.’^35^ *Prima facie*, this view appears to entail the rationalist interventionist inference that physicians need to seek to remedy the kinds of irrationality that are apparent in the hopes involved in examples (a), (b), and (d).

But, despite appearances, the above considerations do not actually entail the rationalist interventionist view. To see why, it is helpful to draw a comparison between hope and certain kinds of conviction grounded in religious faith. As Robert Audi has pointed out, having faith that P does not always connote the belief that P is true; indeed, he notes that ‘one can have faith that a friend will survive cancer, without either believing or disbelieving this.’^36^ This suggests that items of faith‐based conviction need not function as straightforward beliefs. Consider that on one plausible account, the primary goal of a belief is to aim at the truth.^37^ Conversely, items of faith‐based conviction need not have truth as their primary aim; instead, items of faith‐based conviction may incorporate sub‐belief components with other aims (such as the aim of showing religious commitment) that are not subject to the same norms of rationality as are standard beliefs. Furthermore, individuals might *choose* to have items of faith, for the purpose of achieving a religious goal (such as coming to regard themselves as having a relationship with a supernatural being), in a way that they cannot rationally choose to have non‐religious beliefs.^38^ To put the point another way, it might be quite possible for people to be *practically* rational in choosing to have a faith‐based conviction that P will probably occur, even if it would be theoretically irrational to believe that.^39^ Because we hold that patients ought to be permitted to act on considerations of practical rationality (pertaining to what we have reasons to do), as well as theoretical rationality (pertaining to what we have reasons to believe), we refer to our position as the practical rational view.

Of course, in some cases, the cognitive component of hope may simply amount to a straightforward irrational belief. In these cases, on our practical rational view, physicians have a *prima facie* duty to try to prevent action on the basis of patient decision‐making that is straightforwardly irrational. Note however, that in complex cases of hope, it will not always be easy to distinguish the expression of practical rationality from expressions of theoretical irrationality. Indeed, the adoption of a hopeful attitude that exhibits theoretical irrationality may be an expression of the patient's autonomous commitment to a religious value or way of life. To remedy that irrationality would require the unjustified assumption that the physician's duty to facilitate the patient's local autonomous decision‐making outweighs their duty to respect the patient's overall autonomy. We thus advocate a broader view than the rationalist interventionist view. On our practical rational view, to determine whether the hope in question is compatible with autonomous choice, physicians must ascertain whether that hope incorporates a truly irrational belief or whether it instead amounts to a manifestation of the patient's autonomous commitment to a way of life, which achieves a religious goal (broadly understood), and so counts as an expression of practical rationality.

## 5 SECTION 5

Having outlined the case for the practical rational view, we will now explain its implications for our four exemplar cases of conviction‐based hope in health care. Concern about a patient's hope impeding their understanding of the risks and benefits of medically necessary procedures arises especially in cases (a) and (b). In case (a), Marta's hope for an outcome which conventional medicine holds to be unattainable—that her baby will be born healthy—prevents her from adequately considering issues around her baby's care at birth. Similarly, in case (b), ST's conviction‐based hope in her ability to receive an experimental treatment and in the chance of her benefiting from it prevents her from engaging in discussions about the merits of a more palliative path. In these cases, it will be difficult to obtain the patient's properly informed consent to the medically necessary procedure in question, due to the influence of conviction.^40^ To ameliorate this difficulty, healthcare professionals can attempt to redirect their patient's conviction‐based hope to bring it closer in line with the hope that a patient ought to hold, by the lights of medicine (both in terms of its goal and its intensity).

Healthcare professionals could encourage Marta and ST to prepare for the worst (that the baby will not be born healthy and will die soon after birth, or that ST will further deteriorate before the experimental treatment is possible), while still hoping for the best. Healthcare professionals could explain to both Marta and ST that maintaining hope and remaining positive can be consistent with practical preparation for other contingencies.

Concern about a patient's hope impeding their understanding of risks and benefits of medically necessary procedures is less applicable to cases (c) and (d). In case (c), the Amish man's conviction‐based hope that the traditional herbal ointments and burdock leaf dressing would (along with conventional antibiotics) successfully and inexpensively treat his burns, to the extent that he was able to return to work safely, did not impede his understanding of the risks involved in declining conventional treatments and skin grafts for his burns. Likewise, in case (d), Helen's unwarrantedly faint hope that a hysterectomy could successfully treat her cancer did not seem to prevent her from acquiring some level of understanding of the health risks involved in declining a hysterectomy at that stage of her cancer. In these types of cases, it is ethically justifiable for healthcare professionals to accept that the patients' hopes are ill‐directed and to seek to mitigate the effects of these.

In case (c), it was made clear to the burns unit staff that the Amish man would not accept medically recommended skin grafts under any circumstances. The burns unit staff accepted this but rightly sought to mitigate the effects of his conviction‐based hope that led him to decline conventional treatment and skin grafts, by negotiating with the Amish elders to allow the man to be treated with antibiotics. By going along with the Amish man's traditional treatment and giving up on the medically recommended outcome of reduced scarring, the burns unit staff were able to ensure that the Amish man attained a desirable outcome. Although his key priority (being able to return to work) was different from the standard goal (minimising the severity of damage from burns), his priority is not inconsistent with the ordinary goals of health care. Indeed, it can be regarded as an expression of practical rationality. In exhibiting an autonomous commitment to a way of life, he helps achieve a practical personal goal, which is grounded in religious conviction.

In case (d), Helen eventually agreed to have a hysterectomy to alleviate pain. Perhaps healthcare professionals could have engaged with Helen's fatalistic reasoning earlier, for example, by advising her that ‘God helps those who help themselves.’^41^ Alternatively, if Helen were pessimistically concerned about the potential downsides of ‘getting her hopes up,’ healthcare professionals might reassure her that, as Vaclav Havel commented: ‘Hope isn't optimism which expects things to turn out well, but the belief that there is still good worth working for.’^42^

People often have a variety of hopes, not all of which guide their decisions and actions. A patient's guiding or core hopes can be understood as akin to the tip of an arrow, directed at a particular target which they are (actively) seeking to attain, while their other hopes are usually more peripheral and may not influence their actions.^43^ If they are to attempt to redirect, adjust, or supplement patients' conviction‐based hopes (as in cases (a) and (b)), healthcare professionals attempts will have to focus only on those hopes which guide or influence patients' decisions and actions. (It would be objectionably intrusive for healthcare professionals to attempt to redirect or adjust a patient's incidental or peripheral hopes which do not guide the patient's decisions or actions.) And in seeking to mitigate the effects of their patients' ill‐directed hopes in cases such as (c) and (d), healthcare professionals can help patients to accept that a medically plausible outcome is more likely, without purging the patient of their hopes for a very different outcome—provided that those hopes do not undermine the patient's autonomous decision‐making about their medical treatment.^44^

## 6 CONCLUDING REMARK

We have considered four ways in which a patient's convictions (religious or otherwise) can lead them to either have hopes that are aimed at a different goal than the goal of successful medical treatment or to have unrealistic expectations of the chances of a successful treatment, and we have provided practical advice to healthcare professionals about how best to handle such cases. As we have shown, working out how best to handle such cases is far from straightforward. Issues of patient consent, decision‐making capacity, and autonomy need to be taken into account, as does the potential impact of hope on these matters of concern. It is tempting to try to apply a simple rule: either the libertarian's rule that patient choices must always be respected or the rationalist interventionist's rule that only patient decisions that accord with the standards of theoretical rationality need to be respected. However, as we have shown, there is a middle path to be navigated between these unsatisfactory extremes which respects patient choice (provided that it accords with the more relaxed norms of practical rationality). By applying the practical rationality approach that we have argued for here, healthcare professionals can respect conviction‐based hope while also upholding their professional obligations to their patients.^45^

## Footnotes

1 See, for example, Pellegrino, E. D., Thomasma, D. C., & Miller, D. G., (1996). *The christian virtues in medical practice*, Georgetown University Press; and Clarke, S. & Oakley, J. (2024). “Where there's hope, there's life”: On the importance of hope in healthcare. *Journal of Medicine and Philosophy*, jhae 037, 1–12. https://doi.org/10.1093/jmp/jhae037

2 For example, McMillan, J., Walker, S., & Hope, T. (2014). Valuing hope. *Monash Bioethics Review*, *32*, 33–42; Simpson, C. (2004). When hope makes us vulnerable: A discussion of patient‐healthcare provider interactions in the context of hope. *Bioethics*, *18*(5), 428–447.

3 See, Martin, A. (2013). *How we hope: A moral psychology*. Princeton University Press; Snyder, C. R., Rand, K. L. & Sigmon, D. R. (2005). Hope theory. In C. R. Snyder & S. J. Lopez (Eds.). *Handbook of positive psychology* (pp. 257–276). Oxford University Press.

4 We don't mean to imply that other mental states do not also lead to patients obtaining better outcomes. For example, there is evidence that a general sense of optimism helps patients to obtain better healthcare outcomes (Seligman, M. E. (2006). *Learned optimism: How to change your mind and your life*. Vintage, Chapter 10). We understand hope to be directed at particular outcomes, whereas optimism is a more general standing attitude people can have toward one, many or all outcomes. (Also, one can hold out hope that a particular outcome will occur, without being optimistic that this outcome will occur).

5 For extended discussion of the potential harms of false hope, see Eijkholt, M. (2020). Medicine's collision with false hope: The false hope harms argument. *Bioethics*, *34*, 703–711.

6 Wear, S. (1998). *Informed consent: Patient autonomy and clinician beneficence within health care* (2nd ed., pp. 100–125), Georgetown University Press.

7 Back, A. L., Arnold, R. M., & Quill, T. E. (2003). Hope for the best, and prepare for the worst. *Annals of Internal Medicine*, *138*(5), 439–443.

8 Disraeli, B. (1883) [2017]. *The wondrous tale of alroy*. Gale.

9 This phenomenon is often described by clinicians, albeit to our knowledge there are no empirical studies measuring its prevalence.

10 The hopes of healthcare professionals themselves may sometimes lead them, as well as patients, to form unrealistic expectations of the chances of successful treatment, or to aim for different goals than the ordinary goals of healthcare. It would take us too far afield to consider the influence of hope on healthcare professionals here.

11 See, Savulescu, J., & Richard W. M. (1997). Should informed consent be based on rational beliefs? *Journal of Medical Ethics*, *23*(5), 282–88; Walker, R. L. (2009). Respect for rational autonomy. *Kennedy Institute of Ethics Journal*, *19*(4), 339–366.

12 Martin, op. cit. note 3, pp. 7–8.

13 For a recent defence of this venerable account of the aim of healthcare, see Curlin, F. & Tollefson, C. (2021). *The way of medicine: Ethics and the healing profession*. University of Notre Dame Press.

14 Breitbart, W. (2006). The goals of palliative care: Beyond symptom control. *Palliative and Supportive Care*, *4*(1), 1–2.

15 Categories (a) and (b) potentially overlap in the sense that in practice it will often be unclear whether a desired outcome is unattainable or simply unlikely. Nevertheless, it is valuable to distinguish these. Other examples in category (a) include hope for complete recovery in cases of brain death or severe genetic disorders.

16 This is a composite case drawn from the experience of one of the authors whilst working as a neonatologist.

17 See Court of Protection in the United Kingdom. A NHS Trust v ST & Ors [2023] EWCOP 40. A detailed ethical and/or legal analysis of the case is beyond the scope of the paper.

18 Chan, Y. S., Cheng, L. N., Wu, J. H., Chan, E., Kwan, Y. W., Lee, S. M. Y., Leung, G. P. H., Yu, P. H. F., & Chan, S. W. (2011). A review of the pharmacological effects of arctium lappa (burdock). *Inflamopharmacology*, *19*(5), 245–254.

19 Weber, C., Corneman, A., & Dal C. A. (2021). Amish burn treatment meets a major trauma: Centre success with cooperation. *Plastic Surgery Case Studies*, *7*, 1–5.

20 Ibid.

21 Drew, E. M. & Schoenberg, N. E. (2011). Deconstructing fatalism: Ethnographic perspectives on women's decision making about cancer prevention and treatment. *Medical Anthropology Quarterly*, *25*(2), 164–182.

22 Ibid: 169–170.

23 Ibid: 170.

24 Ibid: 169. For discussion of somewhat similar cases, see Franklin, M., Schlundt, D. & Walston, K. (2008). Development and validation of a religious health fatalism measure for the African‐American faith community. *Journal of Health Psychology*, *13*(3), 323–335.

25 See Beauchamp, T. & Childress, J. (2019). *Principles of bioethics* (8th ed.). Oxford University Press.

26 Walker, op. cit. note 11.

27 See Savulescu & Momeyer, op. cit. note 11, p. 283.

28 Ibid: 283.

29 See, for example, Grisso, T., & Paul S. A. (1998). *Assessing competence to consent to treatment: A guide for physicians and other health professionals*. Oxford University Press.

30 See, for example, Pugh, J. (2020). *Autonomy, rationality, and contemporary bioethics*. Oxford University Press.

31 For further discussion of best‐interest assessments, see Birchley, G. (2021). The theorisation of ‘Best Interests’ in bioethical accounts of decision making. *BMC Medical Ethics*, *22*(68), 1–18.

32 With respect to best interest assessments for patients over the age of 16, under Section 4 of the Mental Capacity Act 2005 (MCA), neither the views of the patient herself nor consulted third parties are alone determinative of whether a given treatment is in an individual's best interests.

33 See, under the heading ‘What is Capacity?', the Mental Capacity Act 2005 (MCA): https://www.hra.nhs.uk/planning-and-improving-research/policies-standards-legislation/mental-capacity-act/#:~:text=A%20person%20lacks%20capacity%20if,mental%20illness (accessed 18 June 2024).

34 In ST's case, the judge concluded (contrary to the arguments of the Official Solicitor) that ST was suffering from an impairment of her mind—a consequence of her prolonged critical illness. Of course, even if a patient has capacity, there may yet be other legal justifications for failing to respect a hopeful patient's treatment wishes. For example, doctors are under no legal obligation to provide a futile treatment to a patient simply because the patient hopes that it might work. (Our suggestion is that the court ought to have taken this route in ST's case, though in the actual case a further (substantive) hearing where issues like this might have been aired did not take place as ST died). See: A NHS Trust v ST & Ors [2023] EWCOP 40 (25 August 2023), available at: http://www.bailii.org/ew/cases/EWCOP/2023/40.html (accessed 18 June 2024).

35 Savulescu & Momeyer, op. cit. note 11, p. 282.

36 Audi, R. (2008). Belief, faith, and acceptance. *International Journal for Philosophy of Religion*, *63*(1/3), 87–102.

37 Williams, B. (1973). Deciding to believe. In B. Williams (Ed.), *Problems of the self*, (pp. 136–51). Cambridge University Press.

38 Buckareff, A. A. (2005). Can faith be a doxastic venture? *Religious Studies, 41*(4), 435–445.

39 Pugh, op. cit. note 30.

40 Marta is not necessarily providing informed consent—since she is declining palliative care input. However, her conviction and hope might impact her ability to consent to procedures around the time of delivery (e.g., Caesarean section).

41 This is not to suggest that healthcare professionals working with Helen must themselves believe in God. Nor is it to suggest that they need to be the ones who provides her with this religious advice. Arguably, such advice might more appropriately be provided by a hospital chaplain (see Notini, L. & Oakley, J. (2023). When (if ever) may doctors discuss religion with their patients? *Bioethics, 37*(1), 72–80.

42 See ‘Hope may be in short supply in a world divided by conflict. So what is the alternative?' *ABC News*, 21 December 2022. https://www.abc.net.au/news/2022-12-21/is-hope-useful-in-desperate-circumstances-conflict-big-ideas/101742258 (Accessed 27 November, 2023).

43 For example, the person who ‘holds a candle' to reunite with an ex‐partner but takes no steps to try to bring this about.

44 Note that one can have hope for X, while also having accepted that X is unlikely to occur.

45 For helpful discussions of the issues raised in this paper we are grateful to Alberto Giubilini, Joshua Hordern, Mehrunisha Suleman, Margaret Yee, and feedback from a TORCH Medical Humanities workshop at the University of Oxford as well as from students in the Monash University Master of Bioethics program. This research was funded in part by the Wellcome Trust [203132/Z/16/Z] and [226801/Z/22/Z] and was also supported by Australian Research Council Discovery Project grant DP190101597, Australian Research Council Discovery Project Grant DP240102614 and a Crawford Miller (Oxford‐Australia) Visiting Fellowship.

